# Characterization of a recurrent missense mutation in the forkhead DNA-binding domain of *FOXP1*

**DOI:** 10.1038/s41598-018-34437-0

**Published:** 2018-11-01

**Authors:** Tyler B. Johnson, Keegan Mechels, Ruth Ellen Anderson, Jacob T. Cain, David A. Sturdevant, Stephen Braddock, Hailey Pinz, Mark A. Wilson, Megan Landsverk, Kyle J. Roux, Jill M. Weimer

**Affiliations:** 1 Pediatric and Rare Disease Group, Sanford Research, Sioux Falls, South Dakota USA; 2 Department of Pediatrics, Sanford School of Medicine at the University of South Dakota, Sioux Falls, South Dakota USA; 3 Division of Medical Genetics, Department of Pediatrics, Saint Louis University School of Medicine, Saint Louis, Missouri USA; 4 Department of Biochemistry and Redox Biology Center, University of Nebraska, Lincoln, Nebraska USA

## Abstract

Haploinsufficiency of Forkhead box protein P1 (FOXP1), a highly conserved transcription factor, leads to developmental delay, intellectual disability, autism spectrum disorder, speech delay, and dysmorphic features. Most of the reported FOXP1 mutations occur on the C-terminus of the protein and cluster around to the forkhead domain. All reported *FOXP1* pathogenic variants result in abnormal cellular localization and loss of transcriptional repression activity of the protein product. Here we present three patients with the same *FOXP1* mutation, c.1574G>A (p.R525Q), that results in the characteristic loss of transcription repression activity. This mutation, however, represents the first reported *FOXP1* mutation that does not result in cytoplasmic or nuclear aggregation of the protein but maintains normal nuclear localization.

## Introduction

Haploinsufficiency of forkhead box protein P1 (*FOXP1)* causes a rare neurodevelopmental disorder that is characterized by a vast array of symptoms that include developmental delay, motor coordination deficits, specific language impairment, autistic features, intellectual disabilities, dysmorphic features, various cancers, and congenital defects of the heart, kidneys and urinary tract^1,2^ (MIM 605515 (gene); MIM 613670 (disorder)). The FOX proteins are a family of evolutionarily conserved transcription factors that share a characteristic winged-helix (forkhead) DNA-binding domain. Members of the FOXP subfamily, which includes four paralogous proteins, have crucial roles in embryonic development and organogenesis of heart, lung, esophagus, and immune system^3–8^. Each of these proteins share four common domains: an N-terminal Q-rich (polyglutamine) domain, internal zinc-finger and leucine zipper, and a C-terminal forkhead box DNA-binding domain (FOX).

FOXP1 is widely expressed in human and mouse tissues with specific regional expression in the central nervous system including the cerebral cortex, striatum, and spinal cord^6,9–11^. Its role is crucial in these regions during cortical development for regulation of neuronal migration, differentiation, and morphogenesis^8,12,13^. Suppression of *Foxp1* within the developing brain leads to radial migration defects where neurons improperly localize, as well as problems with morphogenesis and neurite outgrowth^8^. Foxp1 binds a 7-nucleotide consensus sequence, TATTT(G/A)T, and represses transcription at target locations, including SV40 and interleukin-2 promoters^14^, and directly regulates expression of genes important for neurogenesis^12^. FOXP1 also directly binds to and controls expression of genes associated with autism^9^ and abnormalities of the central nervous system, musculature, cognition, and higher mental function^12^. Proteomic studies also show that FOXP proteins directly interact with partners with diverse roles in neurodevelopment including migration of cortical projection neurons, cortical neuron plasticity, and neurogenesis^15^.

Outside the CNS, FOXP1 plays an important role in development monocytes and macrophages^16^, regulatory T-cells^17^, cardiomyocytes^7^, lung epithelial cells^18^. In immune cells, overexpression of FOXP1 in mouse monocytes impaired monocyte differentiation, migration and macrophage function through repression of colony-stimulating factor receptor^16^. Additionally, deletion of *Foxp1* in mice influences B-cell and thymocyte development^3,19^. *Foxp1* also plays an important role in cardiac development as *Foxp1* null mice are embryonic lethal due to defects in myocyte maturation leading to thinning of the ventricular myocardial compact zone^7^.

**Figure 1:**
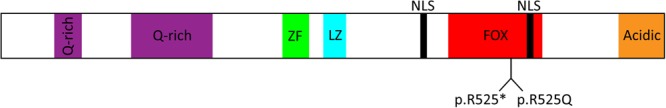
FOXP1 p.R525 mutations map to the FOX DNA-binding domain. FOXP1 protein structure is composed of common conserved domains: an N-terminal Q-rich (polyglutamine) domain, internal zinc-finger (ZF) and leucine zipper (LZ), and a C-terminal forkhead box DNA-binding domain (FOX). Additionally, two nuclear localization signals (NLS) are present at the C-terminus that target the protein to the nucleus under normal conditions. Arginine 525 is localized to the DNA-binding FOX domain nestled between the two NLS.

The native FOXP1 sequence encodes conserved functional domains that dictate its cellular functions (Fig. 1). The N-terminus of the protein contains a polyglutamine (Q-rich) domain, putative zinc finger and leucine zipper domains. Q-rich domains, which are sequences that are commonly present in many transcription factors^20^, have been demonstrated to be important for protein-protein interactions^21^ as well as modulation of transcriptional repression activity^14,22^. Additionally, the zinc finger and leucine zipper contribute to protein-protein interactions and transcriptional repression, as is the case for many other proteins containing these domains^14,23–26^. Through the leucine zipper domain, FOXP1 is able to form specific homo- and heterodimers with its paralog FOXP2^27^. Near the C-terminus are two nuclear localization sequences resulting in the protein’s natural localization to the nucleus in normal tissues and cells^2,28,29^ and a forkhead box DNA-binding domain (FOX); however, exogenously expressed pathogenic variants result in disrupted nuclear localization and formation of cytoplasmic and nuclear aggregates^27,29–31^. Additionally, these mutations result in loss of both transcriptional repression activity and typically the ability to form dimers with wild type FOXP1 and FOXP2^29,31,32^, although there are reported variants that preserve their dimerization capability^29^.

The C-terminal FOX domain, which is critical for its activity as a transcriptional repressor, is the site of more than 80% of all pathogenic missense mutations. The FOX domain is composed of 88 amino acids that are organized into five α-helices and three β-sheets some of which are directly involved in recognition and binding to DNA as well as protein-protein interactions through domain swapping^33^. Siper *et al*. provided a comprehensive discussion on the structural consequences of missense and in-frame mutations occurring in the FOX domain that have been identified in patients^2^. Notably, these mutations all occur on residues important for DNA-binding or protein-protein dimerization. They discuss the implications of changes in amino acid sequence in the FOX domain and note possible obstruction of electrostatic interactions with DNA, DNA recognition, and aberrant protein folding and dimerization.

Several studies have reported a wide spectrum of *de novo* human mutations in *FOXP1* including deletions^34–38^, nonsense and missense variants^1,29,39,40^, frameshifts^30^, and translocations^41^. Here, we present three patients with a missense c.1574G>A (p.R525Q) mutation at the same residue. Although the clinical presentation of patient one has been described in detail^1,39^, the effect of this alteration on FOXP1 localization and activity has never been demonstrated. We provide biochemical characterization of the localization of the protein and a measure of its transcriptional repression activity. Finally, this represents the first reported case of a *FOXP1* missense mutation with normal nuclear localization and aberrant transcriptional repression.

## Results

### Clinical assessment

The first patient reported to have a *de novo* c.1574G>A (p.R525Q) mutation in *FOXP1* was previously described in two separate reports^1,39^. Briefly, the patient was born to a non-consanguineous couple of Northern European ancestry and had respiratory distress and a left pneumomediastinum shortly after birth. By the age of four, he had presented with horseshoe kidney, hypospadias, urinary tract defects, recurrent fevers, dysmorphic features, global developmental delays, speech delay and autism. He also had undertubulation and shortening of the long bones of the upper and lower extremities as well as episodes of relapsing-remitting fevers.

Patient two presented to the  genetics clinic as an adult with significant intellectual disability including substantial expressive language delay. He had a history of failure to thrive with delayed pubertal development and low testosterone. The patient was normocephalic with a normal skull shape, a tall, sloped forehead, and arched eyebrows with synophrys. He had a normal chin and ears and palate was intact, his teeth were crowded and irregular. He was in constant motion with many self-stimulatory behaviors including kicking, biting, and throwing himself to the ground. He was able to follow directions and carry out tasks but was unable to express himself. An EKG was normal. Whole exome sequencing in a commercial laboratory on the patient and both of his parents revealed the same *de novo FOXP1* variant, c.1574G>A (p.R525Q).

Patient three was born to non-consanguineous parents of mixed European descent. The pregnancy was complicated by spotting, sub chorionic hemorrhage, elevated maternal serum alpha-fetoprotein (MSAFP), possible Dandy-Walker malformation (DWM) with bilateral ventriculomegaly, unilateral ureteral dilation, and fetal macrosomia. He was delivered vaginally at 34 3/7 weeks without complication. The birth weight was 3140 g and length was 46 cm. Apgar scores were 8 and 8, at 1 and 5 minutes, respectively. He was admitted to the neonatal intensive care unit for management of respiratory distress. Hypoglycemia and jaundice were also noted. Postnatal imaging and examination revealed a horseshoe kidney with right hydronephrosis, bilateral undescended testes, and mega cisterna magna versus arachnoid cyst, without evidence of DWM. At approximately six months of life, the patient was diagnosed with sagittal craniosynostosis and macrocephaly. The patient underwent cranial vault reconstruction at age nine months. The patient sat unsupported at 12 months, crawled at 14 months, and walked at 18 months. He was evaluated by child psychology at 1.5 years and was felt to have low average cognition and borderline expressive/receptive language abilities. The patient presented to the genetics clinic at age 2 years 7 months. He did not have any verbal speech, but did know some signs and communicated with pointing at time of initial evaluation. Macrocephaly was noted (OFC 53.8 cm, >99th centile) as well as a broad, tall forehead with a prominent, pointed chin. Ears were well-formed, but low-set. The palate was intact with a mild prominent secondary alveolar ridge and his height was 96 cm (86th percentile). He could run and jump, but was notably clumsy. At the time of the most recent follow up (age 2 years, 11 months) he could say 7 words, knew 6 signs, and was working with a picture system. He could follow multi-step commands. At that time, he could also identify people and colors, and was working to help dress himself. He wore ankle and foot orthoses (AFOs) due to low tone and unsteady gait, as well as a helmet intermittently for protection from falls, although clumsiness was reportedly continuing to improve. The family described the patient to have sensory issues and a bad temper with hitting, biting, and throwing objects when not getting his way. He was working on toilet training. Whole exome sequencing in a commercial laboratory revealed the *FOXP1* c.1574G>A (p.R525Q) mutation. A maternal sample was not available; however, paternal and unaffected full sibling’s samples were negative for the mutation. No additional variants were identified.

### The FOXP1 p.R525Q mutation maintains nuclear localization but loses transcriptional repression

**Figure 2:**
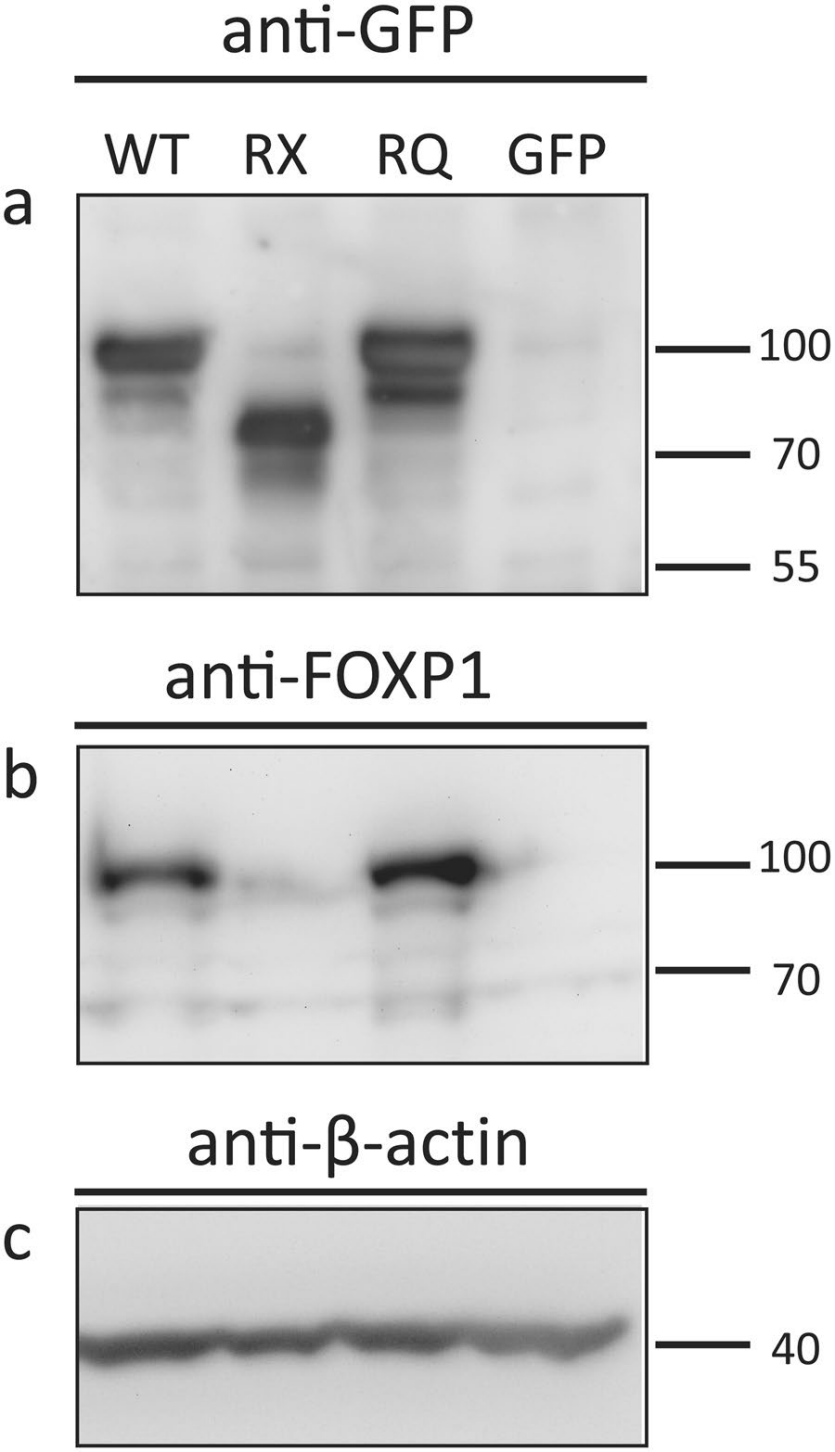
Expression of FOXP1 transcripts in HeLa cells. Expression constructs containing wild type (WT) FOXP1, p.R525* truncating mutant (RX), p.R525Q missense mutant (RQ) or GFP-only control were transiently transfected into HeLa cells. (**a**,**b**) The WT and RQ mutant produce full-length protein while the truncating mutant results in a loss of 152 amino acids at the C-terminus of the protein and a band near 75 kDa. Equal mass was loaded in each lane and (**c**) beta-actin was used as loading control. Blots are cropped to show bands of interest. Full-length blots are presented in Supplementary Figure 1.

**Figure 3:**
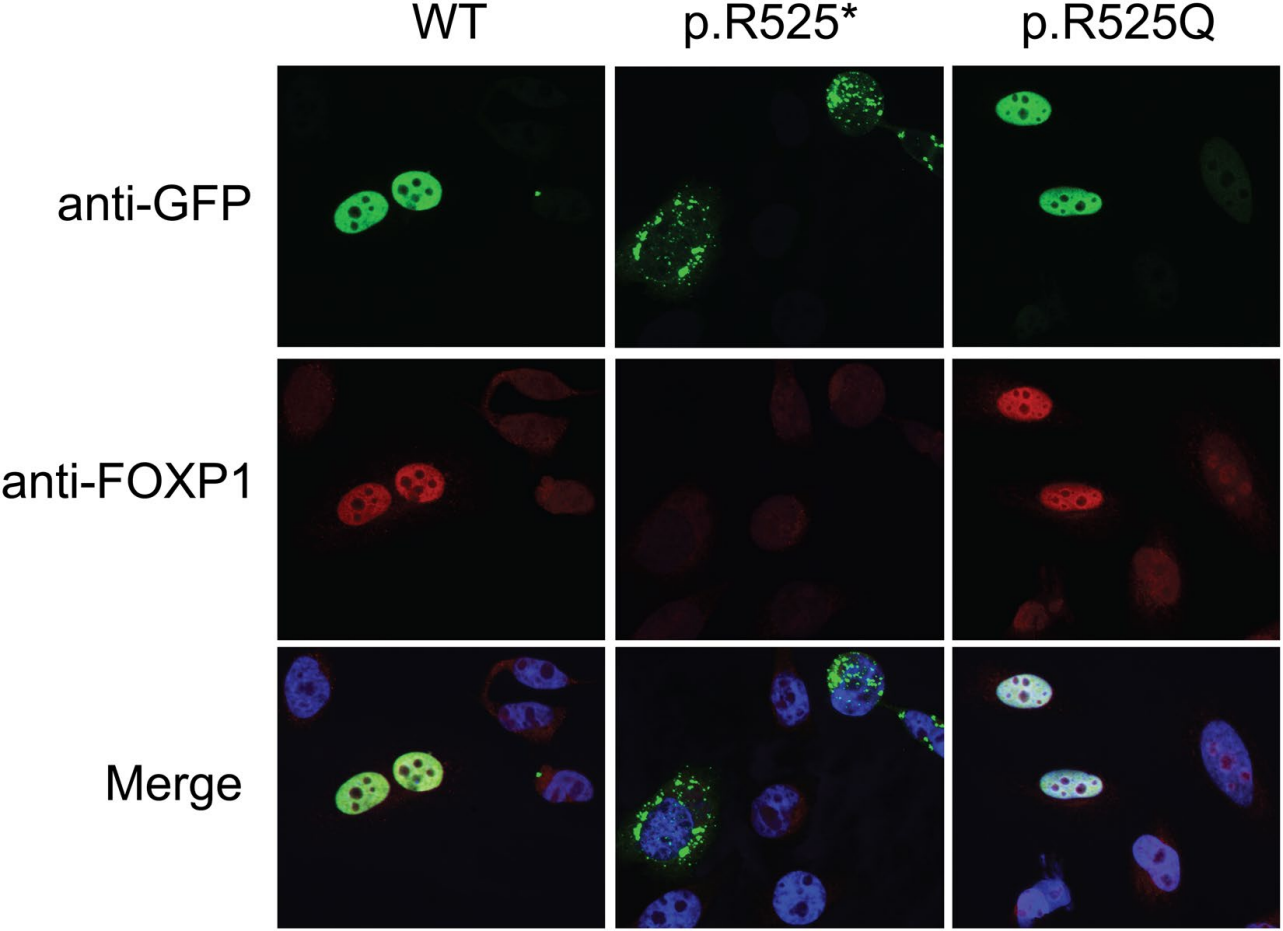
Localization of the FOXP1 proteins in HeLa cells. Expression constructs containing WT FOXP1, truncating p.R525* mutant, and p.R525Q missense mutant were transiently transfected into HeLa cells. WT and p.R525Q mutant properly localize to the nucleus in identical patterns while the p.R525* truncation results in cytoplasmic aggregation.

To determine the effect of the missense p.R525Q on cellular localization, we transiently transfected HeLa cells with constructs overexpressing WT FOXP1, a truncating mutant FOXP1 p.R525*, or the p.R525Q mutant, all fused to an N-terminal GFP reporter (Fig. 2). We performed immunolabeling for the GFP reporter and analyzed the cells by laser scanning confocal microscopy. Wild type FOXP1 localizes to the nucleus like most functional transcription factors (Fig. 3). The truncating mutant (p.R525*) aggregates in the cytoplasm while the missense mutant (p.R525Q) maintained nucleoplasmic localization similar to wild type FOXP1 (Fig. 3).

**Figure 4:**
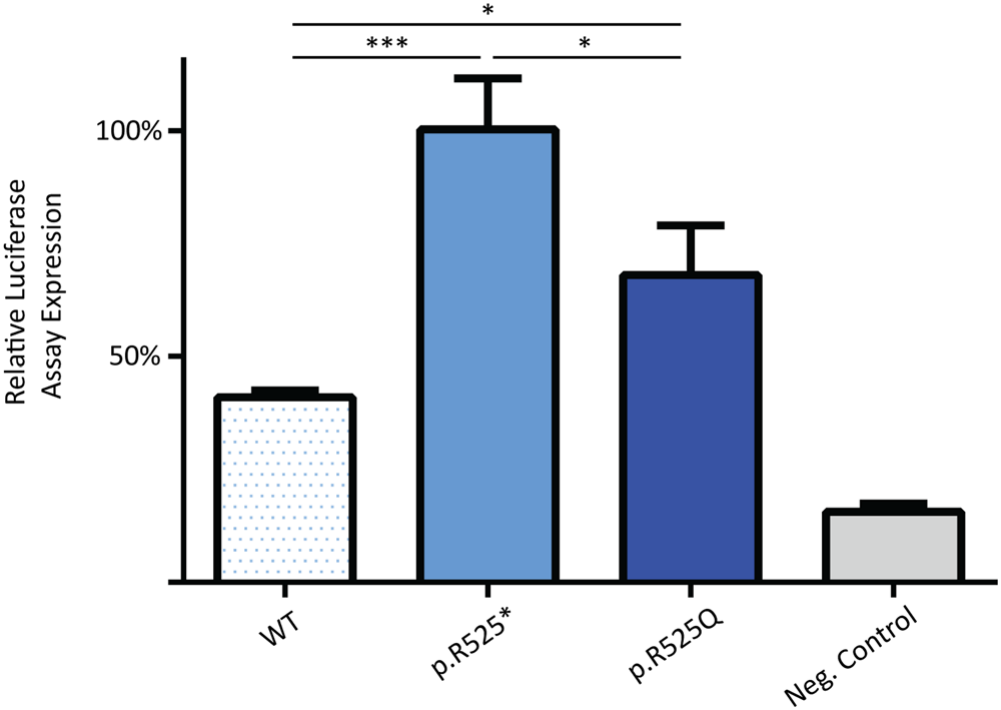
Luciferase activity resulting from transcriptional repression of the SV40 promoter. The bars represent the relative transcriptional activity of the SV40 promoter resulting from co-transfection with plasmids containing the FOXP1 nonsense mutants. The results are mean values ± SEM of experiments performed in technical triplicate. RX and RQ variants result in severely diminished transcriptional repression of the SV40 promoter as compared to WT levels.

Mutations in *FOXP1* result in drastic reduction of SV40 promoter repression *in vitro*^29^. In order to evaluate transcriptional repression activity of the missense mutant compared to wild type and p.R525* levels, we tested the ability of the p.R252Q variant to repress the SV40 promoter. Despite normal nuclear localization of missense mutant, the ability to transcriptionally repress the SV40 promoter was severely diminished as was the case for the truncating mutant (Fig. 4).

## Discussion

Many cases of *de novo* mutations in *FOXP1* have been reported including full and partial gene deletions^34–38^, nonsense and missense variants^1,29,39,40^, frameshifts^30^, and translocations^41^. Shared among the patients harboring heterozygous mutations in *FOXP1* are conditions including intellectual disability, language impairment, autistic features, motor coordination and dysmorphic features also occasionally presenting with various cancers, and congenital defects of the heart, kidneys and urinary tract^1,2^. In this study, we report three patients with a p.R525Q missense mutation in the transcriptional repressor *FOXP1*. These patients all presented with the typical intellectual disability, autism spectrum disorder, language impairment, and behavioral tendencies including aggressiveness and obsessions. However, some of the additional clinical features noted in our patients such as the skeletal anomalies and fevers in patient one and the macrosomia in patient three have not been previously reported in patients with *FOXP1* mutations. The cause of neonatal macrosomia is unknown. Prenatal work ruled out maternal diabetes and there were no other variants identified on whole exome sequencing. The patient did have an 11q14.1 deletion on chromosomal microarray analysis, an intronic deletion of DLG2 that has been identified in one normal control, which was considered to be a variant of unknown significance. Ultimately, we felt the deletion to be noncontributory, and there is certainly no evidence to link it to his history of neonatal macrosomia. Whether the additional features in these patients are a result of the *FOXP1* mutation alone or additional unidentified genetic alterations is yet unclear.

Forkhead box proteins are a highly conserved class of transcription factors with a diverse role in development and disease. An inherent characteristic of most transcription factors is that they maintain their presence in the nucleus in order to activate or repress the transcription of genes in response to certain stimuli. FOXP1 localization is no exception to this rule, adhering to nuclear localization under normal circumstances^28^. To date, 41 different mutations have been reported in the *FOXP1* gene (Summary in^2^). The Arg525 amino acid appears to be a mutational hotspot as, to date, five additional patients have been reported harboring a p.R525* mutation^2,32,42^. Sollis *et al*. determined that regardless of the mutation, *FOXP1* mutants lost transcriptional repression activity and always resulted in cytoplasmic and/or nuclear aggregation of the resulting protein product, likely due to protein misfolding and loss of NLS sequences or functional loss of NLS motifs^29^. These mutations do not affect the NLS primary sequence but must structurally destabilize the domain sufficiently to impair NLS functionality. Here, the p.R525Q mutant construct, which is predicted to do no more than impair DNA binding, displayed normal nuclear localization. However, we show that the mutation resulted in severely diminished transcriptional repression indicating that DNA-binding was affected by the amino acid substitution while NLS activity is retained.

**Figure 5:**
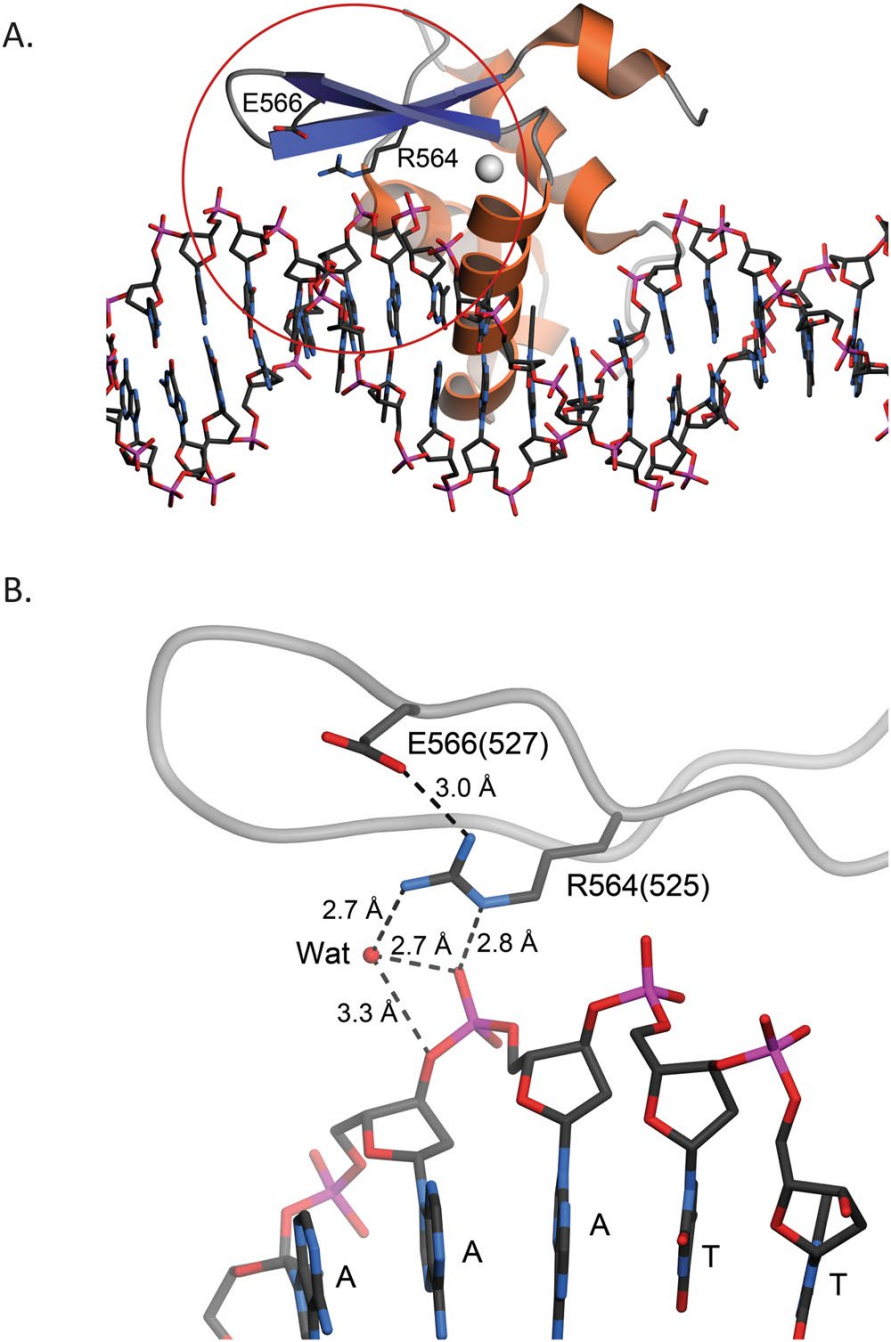
Residue R525 of human FOXP1 is predicted to interact with both DNA and nearby residues. (**A**) Chain K of the forkhead domain of human FOXP2 (PDB 2A07) bound to DNA^43^. DNA is depicted in ball-and-stick and FoxP2 is shown as a ribbon diagram with α-helices colored orange and β-strands colored blue. The forkhead domain of FoxP2 shares 87% sequence identity with human FoxP1. Residue R564 in FOXP2 (corresponding to R525 in FOXP1) is located at the protein-DNA interface. The red circle shows the area that is depicted in panel B. (**B**) A closer view of the environment of R564 (R525 in human FOXP1). Residue numbers in PDB 2A07 are indicated; their corresponding human FOXP1 numbering shown in parentheses. Potential hydrogen bonding interactions are shown in dotted lines with distances indicated in Ångstroms. “Wat” indicates an ordered water molecule mediating H-bonds between protein and DNA. R525 in human FOXP1 is likely to make similar contacts, which the R525Q mutation likely disrupts. Figure made with POVScript+^47^.

The amino acid sequence of the forkhead DNA binding domain of human FOXP1 is 87% identical to the corresponding domain of FOXP2, and thus the structures of these proteins are likely to be highly similar. The 1.9 Å resolution X-ray crystal structure of human FOXP2 in complex with DNA (PDB 2A07^43^) thus provides a useful model for understanding the likely impact of the R525Q mutation in FOXP1. Arg564 of FOXP2 (corresponding to FOXP1 Arg525) makes multiple stabilizing contacts with DNA and other protein elements. The cationic sidechain of Arg564 makes favorable electrostatic interactions with both a backbone phosphate of the target DNA and Glu566. Glu566 is conserved in FOXP1, where it is Glu527 (Fig. 5). We predict that a R525K mutation, which preserves charge, would at least partially rescue the phenotype. However, the guanidinium group of Arg positions three H-bond donors about ~2.3 Å apart, which is ideal for making multiple H-bonds with anionic acceptors such as carboxylate side chains or phosphates. The structure of Foxp2 shows that R564 makes multiple H-bonds that include the phosphate backbone, a proximal Glu, and an ordered water. Lysine is a more limited H-bond donor, and a Lys at this position could not satisfy all of these H-bond acceptors simultaneously. Therefore, we expect that the R525K phenotype would be intermediate between the R525Q and wild-type ones. Although this Arg residue is within ~10 Å of the dimer interface in PDB 2A07, it does not make any dimer-spanning contacts. It is difficult to strictly rule out dimer destabilization without experimentally assessing this, but the structure of the homologous FOXP2 indicates that dimer disruption is not likely to be the primary effect of the R->Q substitution at this location. Additionally, we cannot exclude alteration in any known FOXP1 protein binding partners as the only structures available for analysis are DNA-bound FoxP2, not complexes with other proteins. Within the limits of what is available for us to analyze, the R525 environment is dominated by DNA-protein and intrachain protein contacts that would make protein-protein contacts less likely without a major conformational change. Therefore, the clinical p.R525Q mutation in FOXP1 likely destabilizes both intra-protein and protein-DNA interactions due to loss of the cationic guanidinium sidechain of Arg525.

In summary, we have shown that three patients with the same missense mutation (p.R525Q) in the forkhead box DNA-binding domain of *FOXP1* results in global developmental delay, autism spectrum disorder, and characteristic dysmorphic features. Although other variant *FOXP1* mutations result in the same clinical presentation resulting from loss of transcriptional repression activity and protein aggregation, the p.R525Q mutation maintains normal nuclear localization as the mutation is predicted to only disrupt protein-DNA complexes rather than its NLS signals. Thus, small changes in the DNA-binding domain of FOXP1 that alter electrostatic interactions with DNA and proteins appear to severely affect its function despite translation of full-length protein. Future work comparing cellular transcriptomes of p.R525Q, p.R525*, and other FOXP1 variants would identify some of the shared fundamental changes among these mutations that lead to a similar clinical phenotype in patients.

## Methods

### Plasmid generation

*FOXP1* genes were amplified from human cDNA (HsCD00297105, ORFeome Collaboration, Harvard PlasmID Database) and inserted into pcDNA3.1 (Addgene, Cat. No. #) for N-terminal fusion with GFP by In-Fusion cloning (Clontech). The GFP pcDNA3.1 was linearized by digestion with XhoI and AflII. Primers pairs designed to contain a 15 bp homologous region to the flanking sequences of pcDNA3.1 included 5′-GACGAGCTGTACAAGGACCTCGAGATGCAAGAATCTGGG-3′ & 5′-GATCAGCGGTTTAAACTTAAGCTACTCCATGTCCTCGTT-3′ used to amplify the *FOXP1* WT insert; and 5′-GACGAGCTGTACAAGGACCTCGAGATGCAAGAATCTGGG-3′ & 5′-GATCAGCG GTTTAAACTTAAGTCACACAAAACACTTGTG-3′ used for the p.R525X truncating mutation insert. A three-piece In-fusion cloning strategy was used to generate the p.R525Q plasmid with primers spanning the missense mutation (underlined) as follows 5′ GACGAGCTGTACAAGGACCTCGAGAT GCAAGAATCTGGG-3′ & 5′-TTAACGTTTTCTACTTGC ACAAAACACTTG-3′; and subsequently 5′-CAAGTGTTTTGTGCAAGTAGAAAACGTTAA-3′& 5′-GATCAGCGGTTTAAACTTAAGCTAC TCCATGTCCTCGTT-3′ to generate products of 472 and 1586 bps respectively. The insert to vector molar ratio for HD recombination was approximately 3:1 or 2:2:1. All PCR reactions used CloneAmp HiFi PCR Premix (Takara Bio USA, Cat. No. 639298) as per manufacturer’s recommendations with an annealing temperature of 70 °C. Insertion of gene sequences was confirmed by Sanger sequencing.

### Western blot

Cell Lysates were collected in 200 ul of Laemmli Buffer (BioRad), heated to 98 C for five minutes, and sonicated. 10 ul of lysate was ran on precast gradient gels, 4–20%, and then transferred to a nitrocellulose membrane using the BioRad Turbo Transfer System. Primary antibody incubation was performed with rabbit anti-FOXP1 (Abcam; ab16645^44^) or chicken anti-GFP antbodies (Abcam; ab13970^45^), followed by a secondary antibody incubation using goat anti-rabbit HRP and goat anti-chicken HRP antibodies (Santa Cruz), respectively. Bands were then visualized with Luminata Forte Western HRP substrate (Millipore) and imaged using UVP-gel documentation system.

### Immunocytochemistry

Transient transfections were carried out as previously described^46^. Briefly, HeLa cells were grown in 24 well plates on coverglass to 60–80% confluency. Cells were transfected with 500 ng pcDNA 3.1_FoxP1 WT, p.R525*, or p.R525Q separately using Lipofectamine 3000 reagents (Thermo Fisher Scientific) following the manufacturer’s suggested protocol. After 48 hours, cells were fixed with 3% paraformaldehyde for 20 minutes. Primary antibody incubation was performed with rabbit anti-FOXP1 (Abcam) and chicken anti-GFP antibodies (Abcam) followed by a secondary antibody incubation using anti-rabbit-568, anti-chicken-488 (Invitrogen), and DAPI (Life Technologies). Imaging for cellular localization was carried out on a Nikon Ni-E microscope equipped with a Photometrics CoolSNAP Myo camera. Exposures for each channel were 700 ms (DAPI), 900 ms (GFP), and 300 ms (Texas Red).

### Luciferase assay

HEK 293 cells were grown in DMEM (Corning) supplemented with 10% Fetal Bovine Serum (VWR) and 1% Penicillin/Streptomycin (HyClone) in 24 well plates. When the cells reached 80% confluence, co-transfection of pcDNA3.1-*FOXP1* (250 ng), DBH-pGL3-basic (250 ng), and pRL-TK (25 ng, Promega) was done using Lipofectamine 2000 (ThermoFisher) following the manufacturer’s suggested protocol. After 24 hours, fresh DMEM was added and 24 hours later the cells were lysed using 1X Passive lysis buffer (Promega). Firefly and Renilla luciferase activity was detected using the Dual-Luciferase Reporter Assay System (Promega) following the manufacturer’s suggested protocol. Luciferase activity readings were acquired on biological triplicates and technical triplicates using a Promega GlowMax 96 well luminometer (Promega). 1-way ANOVA with Fisher’s LSD test were used for statistical analysis.

## Electronic supplementary material

Supplementary Information

## Data Availability

The datasets generated during and/or analysed during the current study are available from the corresponding authors on reasonable request.
