# Author Correction: Characterization of a recurrent missense mutation in the forkhead DNA-binding domain of *FOXP1*

**DOI:** 10.1038/s41598-020-62950-8

**Published:** 2020-04-15

**Authors:** Tyler B. Johnson, Keegan Mechels, Ruthellen H. Anderson, Jacob T. Cain, David A. Sturdevant, Stephen Braddock, Hailey Pinz, Mark A. Wilson, Megan Landsverk, Kyle J. Roux, Jill M. Weimer

**Affiliations:** 1 Pediatric and Rare Disease Group, Sanford Research, Sioux Falls, South Dakota USA; 2 Department of Pediatrics, Sanford School of Medicine at the University of South Dakota, Sioux Falls, South Dakota USA; 3 Division of Medical Genetics, Department of Pediatrics, Saint Louis University School of Medicine, Saint Louis, Missouri USA; 4 Department of Biochemistry and Redox Biology Center, University of Nebraska, Lincoln, Nebraska USA

Correction to: *Scientific Reports* 10.1038/s41598-018-34437-0, published online 01 November 2018

The original version of this Article contained an error in the name of author Ruthellen H. Anderson, which was incorrectly given as Ruth Ellen Anderson. This has now been corrected in the PDF and HTML versions of the Article, and in the accompanying Supplementary Information file.

