# Supplementary material for: Characterization of a recurrent missense mutation in the forkhead DNA-binding domain of FOXP1
Source: Sci Rep. 2018 Nov 1;8:16161. doi: 10.1038/s41598-018-34437-0 (PMC6212433; doi:10.1038/s41598-018-34437-0)
Supplement: Supplementary file 1 — Supplementary Information [file 41598_2018_34437_MOESM1_ESM.pdf]

Characterization of a recurrent missense mutation in the forkhead DNA-binding domain of *FOXP1*

Tyler B. Johnson[1], Keegan Mechels[1], Ruth Ellen Anderson[1], Jacob T. Cain[1], David A. Sturdevant[1], Stephen Braddock[3], Hailey Pinz[3], Mark A. Wilson[4], Megan Landsverk[2], Kyle J. Roux[1,2*], Jill M. Weimer[1,2*]

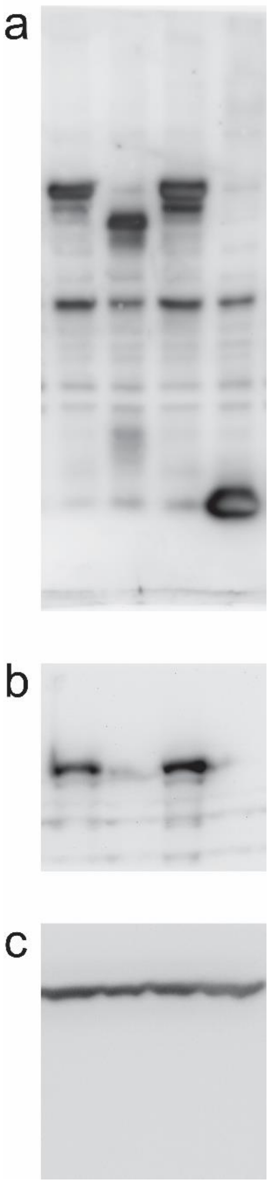

Supplementary Figure 1: Expression of FOXP1 transcripts in HeLa cells. Full length blots from Figure 2 in text.
